# Intertumoral Heterogeneity of Primary Breast Tumors and Synchronous Axillary Lymph Node Metastases Reflected in IHC-Assessed Expression of Routine and Nonstandard Biomarkers

**DOI:** 10.3389/fonc.2021.660318

**Published:** 2021-11-03

**Authors:** Wojciech Kuncman, Magdalena Orzechowska, Łukasz Kuncman, Radzisław Kordek, Katarzyna Taran

**Affiliations:** ^1^ Department of Pathology, Medical University of Łódź, Łódź, Poland; ^2^ Department of Molecular Carcinogenesis, Medical University of Łódź, Łódź, Poland; ^3^ Department of Radiotherapy, Medical University of Łódź, Łódź, Poland; ^4^ Laboratory of Isotopic Fractionation in Pathological Processes, Department of Pathomorphology, Medical University of Łódź, Łódź, Poland

**Keywords:** breast carcinoma, metastases, axillary lymph node, IHC, prognosis, intertumoral heterogeneity

## Abstract

Breast cancer (BC) remains a significant healthcare challenge. Routinely, the treatment strategy is determined by immunohistochemistry (IHC)-based assessment of the key proteins such as estrogen receptor (ER), progesterone receptor (PR), human epidermal growth factor receptor 2 (HER2), and Ki-67. However, it is estimated that over 75% of deaths result from metastatic tumors, indicating a need to develop more accurate protocols for intertumoral heterogeneity assessment and their consequences on prognosis. Therefore, the aim of this preliminary study was the identification of the expression profiles of routinely used biomarkers (ER, PR, HER2, Ki-67) and additional relevant proteins [Bcl-2, cyclin D1, E-cadherin, Snail+Slug, gross cystic disease fluid protein 15 (GCDFP-15), programmed death receptor 1 (PD-L1), and phosphatase of regenerating liver 3 (PRL-3)] in breast primary tumors (PTs) and paired synchronous axillary lymph node (ALN) metastases. A total of 67 tissue samples met the inclusion criteria for the study. The expression status of biomarkers was assessed in PTs and ALN metastases using tissue microarrays followed by IHC. In 11 cases, the shift of intrinsic molecular BC subtype was noticed between PTs and paired ALN metastases. Moreover, a significant disproportion in E-cadherin presence (p = 0.0002) was noted in both foci, and the expression status of all proteins except for HER2 demonstrated considerable variance (k = 1, p < 0.0001). Importantly, in around 30% of cases, the ALN metastases demonstrated discordance, i.e., loss/gain of expression, compared to the PTs. Intertumoral synchronous heterogeneity in both foci (primary tumor and node metastasis) is an essential phenomenon affecting the clinical subtype and characteristics of BC. Furthermore, a greater understanding of this event could potentially improve therapeutic efficacy.

## Introduction

Breast carcinoma (BC) remains a healthcare challenge of high importance. Each year, over 1,350,000 new cases are reported, with a mortality rate exceeding 500,000 (1, 2). Although the mortality of BC remains stable, despite its increasing incidence, over 75% of deaths are caused by metastatic tumors that may express a different profile of clinically relevant biomarkers, such as estrogen receptor (ER), progesterone receptor (PR), and HER2, than the primary mass (3). Thus, the major challenges originate from BC complexity of percolating genomic landscapes and compositional intratumoral and intertumoral heterogeneity reflected by the clinical behavior of breast tumors, as has been emphasized by Ellsworth et al. (4). This compositional diversity within a tumor arises from the clonal selection driven by an acquired set of somatic mutations, and this plays a critical role in the initial diagnosis and the choice of a treatment strategy. However, intertumoral heterogeneity, particularly that observed between primary and metastatic tumors, has recently acquired growing significance in the management of BC.

The identification of five intrinsic molecular subtypes of BC involving genetic patterns of expression by Perou et al. (5) was a milestone in precision medicine and revealed the various roads of carcinogenesis. However, high costs, lack of public access to advanced technologies such as microarrays or sequencing, and difficulties in interpretation of the results still remain as the insurmountable obstacles to apply such a classification system as a clinical standard. Instead, the routine prognostication and treatment decisions are made upon simplified surrogate protocols described in the European Society for Medical Oncology (ESMO) guidelines, which are a gold standard in the clinical management of BC (6). The simplification of the molecular classification (i.e., BC intrinsic subtypes) of Perou et al. (5) that makes it suitable for clinical use involves grouping of the tumors into surrogate intrinsic subtypes [hereinafter called luminal A-like, luminal B-like, HER2-positive (non-luminal), and triple-negative—extensively described in the ESMO guidelines], which are defined only by routine immunohistochemistry (IHC)-based assessment of the key proteins such as ER, PR, HER2, and Ki-67, as well as fluorescence *in situ* hybridization (FISH) assessment of HER2 in vague cases (2+ by IHC). These molecular features, together with clinical factors, have a significant influence on the benefits achievable from specific therapies (7–9). However, IHC still raises controversy due to the subjective assessment of the pathologist, which makes it ambiguous, and intratumoral heterogeneity leading to unprecise recommendations for BC classification (i.e., no recommended cutoff for Ki-67 in ESMO guidelines). Moreover, the assessment is restricted to the primary tumor (PT) only of the 2–3-mm diameter preoperative oligobiopsy (biospecimen), commonly ignoring the protein profile of the postoperative tissue or synchronous axillary lymph node (ALN) metastases. It is also unclear whether these profiles overlap or are different one from the other and how these profile changes may affect prognosis. All these concerns constitute thus the weaknesses of the current practices and raise the unreliability of clinically used techniques beyond the phenomenon of tumor heterogeneity *per se*.

This study is therefore a prelude to reveal the poor efficiency of current protocols and to emphasize the strong need to develop more accurate guidelines for prognostication and treatment decision-making process in BC. Thus, we present the preliminary study that was designed to replicate the conditions of routine assessment of BC biospecimens collected before surgery. For this purpose, we identified the profiles of expression of routinely used biomarkers (ER, PR, HER2, and Ki-67) and other proteins that play potential roles in BC carcinogenesis (Bcl-2, cyclin D1, E-cadherin, Snail and Slug, GCDFP-15, PD-L1, and PRL-3) in the PTs and paired ALN metastases to demonstrate that such assessment may be insufficient to the potential detriment of the patients.

Bcl-2 is an antiapoptotic protein, whose expression unexpectedly correlates with improved overall survival and disease-free survival in BC mainly among luminal A-like breast tumors that have retained ERα signaling (10). Therefore, the assessment of Bcl-2 expression could be advantageous in the substratification of luminal A-like tumors retaining ER activity from those with positive ER status but with inactive ER signaling.

Cyclin D1, a major participant in the cell cycle, is considered a marker of the mitotic phase (11). Its overexpression has been revealed in approximately 50% of BCs, although the unfavorable consequences were noted only among luminal tumors (12), especially with simultaneous amplification of the *CCND1* gene (13, 14).

E-cadherin is an adhesive molecule participating in epithelial-to-mesenchymal transition (EMT). The loss of E-cadherin results in the acquisition of the ability to migrate by the tumor cells and thus progressing disease (15). In the microscopic assessment, the loss of E-cadherin is associated with lobular histologic subtype (different types of stromal infiltration), which in turn is associated with more frequent recurrence, worse prognosis, and increased resistance to chemo- or radiotherapy (16). However, these findings do not entail the use of modified treatment standards.

Snail and Slug, encoded by *SNAI1* and *SNAI2*, belong to the family of transcription factors triggering EMT (17). The transition of the cellular phenotype into mesenchymal expressing Snail or Slug has been associated with unfavorable prognosis in BC, which is commonly accompanied by the downregulation of claudin-1, a major constituent of the tight junction complexes; it was hence concluded that the claudin-low BC subtype is in fact a tumor manifesting mesenchymal features with Snail and Slug overexpression (18).

GCDFP-15 is primarily found in normal breast tissue. In clinical practice, an IHC confirmation of GCDFP-15 combined with BC morphology indicates tumor origin from the primary site (19, 20).

PD-L1 is a member of the immunoglobulin family deactivating the immune response targeted toward tumor cells. Its significance has been confirmed among advanced triple-negative BCs, with higher PD-L1 expression and more numerous tumor-infiltrating lymphocytes (TILs). These findings are reflected in the pembrolizumab-based anti-PD-L1 immunotherapy; this approach has recently been approved by the US Food and Drug Administration (FDA) in combination with chemotherapy to treat unresectable locally advanced or metastatic triple-negative, PD-L1-positive BC (21). However, the clinical relevance in other BC subtypes remains unclear.

PRL-3 is a tyrosine phosphatase regulating the cell cycle, growth, differentiation, and tumorigenesis and is found in 62%–75% BC cases. PRL-3 usually correlates with a worse prognosis and increased risk of metastasis (22); it is believed to take part in the metastasis process of BCs, thought to be the major cause of BC-related deaths (3, 23).

## Methods

### Patients

A total of 67 female patients with histologically confirmed Union for International Cancer Control (UICC) TNM (ver. 7) stage T1-T4N1-2M0 BC qualified for surgery were eligible for the present study. The exclusion criteria for all participants included the presence of microcarcinomas (pT1mi, diameter <1 mm), micrometastasis in lymph nodes (pN1mi, diameter <2 mm), extensive necrosis, the coexistence of other carcinomas, or intensive inflammatory infiltration. The cohort characteristics are shown in **Table 1**.

**Table 1:** Detailed characteristics of the study cohort.

	Size (n = 67)
Age	
- median (range)	70 (40–94)
- mean ± SD	71 ± 14
Histological type*	
- infiltrating duct carcinoma (NOS), 8500/3	66
- lobular carcinoma NOS, 8520/3	1
Grade	
- G2	35
- G3	30
- Gx**	2

*Based on WHO Breast General Classification (24).

**Cannot be determined due to neoadjuvant treatment administration. SD, standard deviation, NOS, not otherwise specified.

### Construction of Tissue Microarrays and Immunohistochemistry

All collected BC resections were matched with their corresponding ALN dissections, followed by the preparation of tissue microarrays (TMAs) using the Sakura Tissue-Tek Quick-Ray system and immunostaining. In the present study, one core measuring 3 mm was taken from the PTs and ALN metastases; this provided sufficient core area to determine the marker expression and replicate the conditions of routine assessment of presurgical biopsy specimens. Moreover, due to the intratumoral heterogeneity, especially regarding Ki-67 and PR, the hematoxylin–eosin (HE)-stained specimens were assessed prior to the core sampling to localize hotspot compartments of non-necrotic high cellular concentration.

The expression profiles of ER, PR, Ki-67, HER2, E-cadherin, cyclin D1, Bcl-2, GCDFP-15, Snail+Slug, PD-L1, and PRL-3 (PTP4A3) in the primary BC tumors and matched synchronous ALN metastases were determined using IHC. The procedure was performed using standard manufacturer protocols regarding the appropriate antigens. Briefly, each TMA section was dewaxed using xylene and rehydrated with concentrated ethyl alcohol. Antigens were retrieved by heat-induced epitope retrieval (HIER) (EnVision FLEX Target Retrieval Solution and EnVision FLEX Wash Buffer). Sections were also blocked for endogenous peroxidase using Dako Peroxidase Blocking Reagent followed by immunostaining (Dako Autostainer, Dako EnVision FLEX) according to the manufacturer’s protocol. Finally, the slides were counterstained in hematoxylin for 3 min. A more detailed description of the IHC procedure, including used antibody clones, manufacturer, dilutions, and incubation times, is given in **Supplementary Table S1**. All of the results followed the ESMO guidelines and comprised the following measures: percentage of ER-, PR-, and Ki-67-positive cell nuclei (index) and the expression intensity of ER, PR, and HER2. Allred score (25) for ER and PR was counted. Cyclin D1, Bcl-2, GCDFP-15, Snail+Slug, and PRL-3 were scored on a 0/1+/2+/3+ scale, and the presence or absence of E-cadherin and PD-L1 (binary outcome) was assessed. The BC subtype and the shift in subtypes between PT and matched ALN metastasis was determined according to the surrogate intrinsic subtypes, as is recommended by the ESMO guidelines (6), hereinafter called luminal A-like, luminal B-like, and “other” gathering the non-luminal subtypes (HER2-enriched, triple-negative).

### Manual Evaluation of Specimens and Statistical Analysis

The prepared slides were coded and assessed by two independent pathologists. Divergent results were additionally consulted. All assessments were performed blind, with no possibility of linking the primary tumor to the matching ALN metastasis sample.

**Figure 1:**
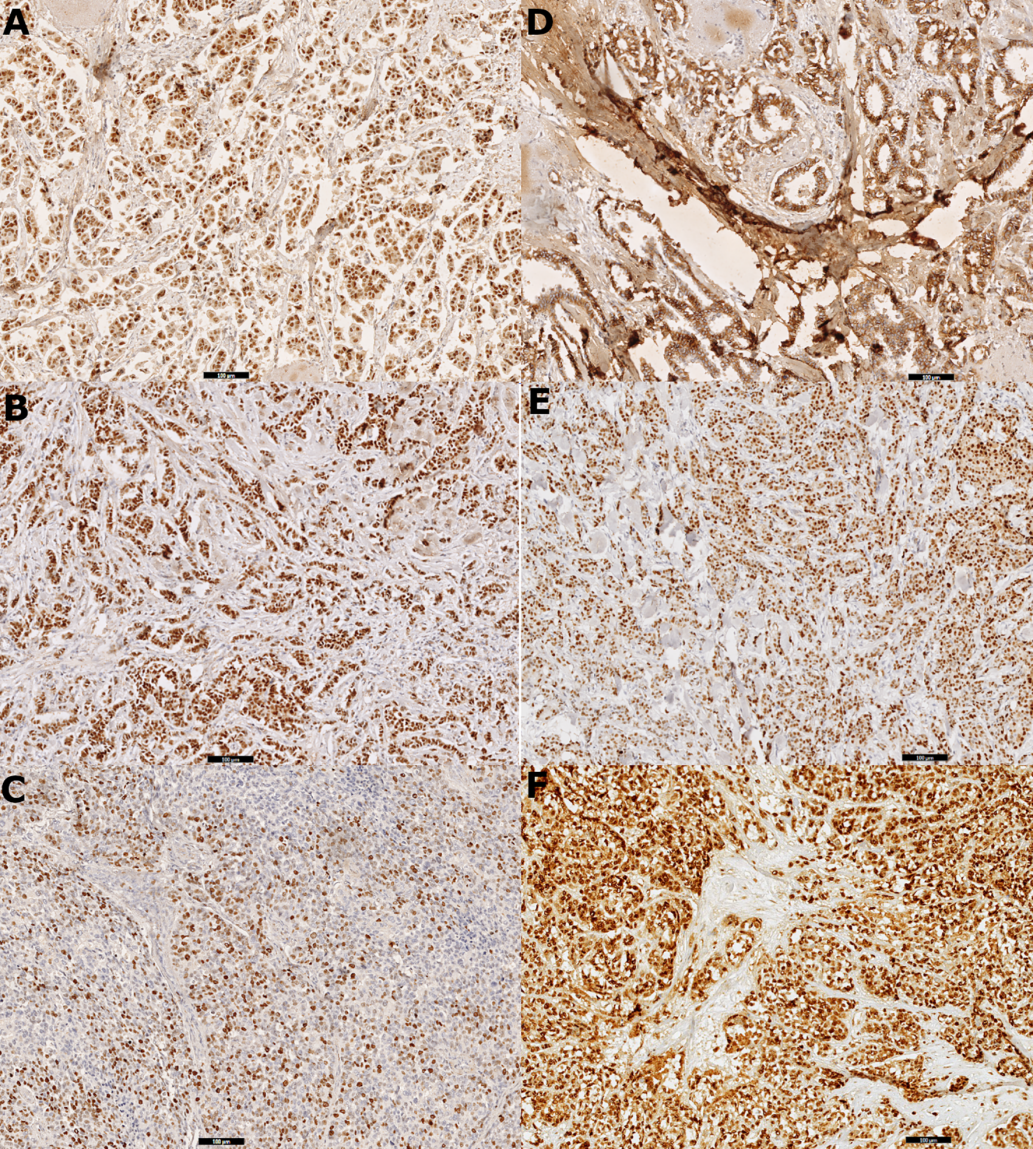
Digital scans presenting **(A)** strong positive (3+) immunohistochemistry staining for estrogen receptor (ER) in 100% cells in primary tumor tissue, **(B)** strong positive (3+) immunohistochemistry staining for progesterone receptor (PR) in 100% cells in primary tumor tissue, **(C)** Ki-67 immunohistochemistry staining: 80% positive tumor cells in node metastasis, **(D)** positive immunohistochemistry staining for E-cadherin in primary tumor tissue, **(E)** strong positive (3+) immunohistochemistry staining for cyclin D1 in 90% cells in primary tumor tissue, and **(F)** strong positive (3+) immunohistochemistry staining for Snail+Slug (mix) in 100% cells in primary tumor tissue.

The cutoff thresholds for positive expression based on the index of positive cell nuclei were set as follows: ≥1% for ER, ≥20% for PR, and ≥16% for Ki-67 (Ki-67 cutoff was defined based on the laboratory median of expression). The HER2-positive cases were defined using the expression intensity, with a 3+ score as positive and 0/1+ as negatives; a 2+ score was excluded from considerations, as it requires confirmation *via* FISH. For the remaining biomarkers (cyclin D1, Bcl-2, GCDFP-15, Snail+Slug, and PRL-3), any level of intensity (1+/2+/3+) was regarded as positive. **Figure 1** presents spots of digital scans of the representative IHC stainings of chosen biomarkers (whole-slide imaging technology), whereas all scans showing staining scores are enclosed as the Supplementary Figures.

The expression status of the ER, PR, HER2, E-cadherin, cyclin D1, Bcl-2, GCDFP-15, Snail+Slug, PD-L1, and PRL-3 was compared between the PT and synchronous matched ALN metastases using the χ^2^ or Fisher’s exact test, according to the expected values. The inter-raters Cohen’s kappa reliability analysis (26, 27) was used to determine the magnitude of the agreement for all proteins between PTs and matched ALN lesions. Any non-normally distributed indices associated with ER- and PR-positive cells, as well as ER/PR Allred scoring between the two groups, were compared using the non-parametric Wilcoxon signed-rank test for paired samples. Differences of p < 0.05 were considered statistically significant.

The multiple correspondence analysis (MCA) is a type of dimensional multivariate exploratory data analysis [method related to the principal component analysis (PCA)] that allows analyzing the set of categorical type variables that describe the individuals. PTs and matched ALN metastases were subjected to dimensional partitioning involving the resultant expression of all markers based on MCA for 37 primary and 15 ALN tumors with the complete set of observations to confirm their divergent overall character. Finally, the associations between Ki-67, ER, and PR indices of positive cell nuclei, the intensity of ER, PR, HER2, Bcl-2, cyclin D1, GCDFP-15, Snail+Slug, and PRL-3 expression, and ER and PR Allred scoring in PTs and ALN metastases were evaluated with Spearman’s rho correlation coefficients; complete sets of paired observations were used from 15 samples. All statistical analyses were carried out in the R 4.0.3 (28) with the dplyr (29), ggpubr (30), FactoMineR (31), factoextra (32), Hmisc (33), and vcd (34) packages.

### Ethical Approval

All procedures performed in this study involving human participants were in accordance with the ethical standards of the institutional research committee and with the 1964 Helsinki Declaration and its later amendments or comparable ethical standards. All participants provided written informed consent prior to enrollment in the study. This study was approved by the Research Ethics Committee of the Medical University of Lodz (approval no. RNN/12/16/KE of January 19, 2016).

## Results

Out of a total of 67 collected specimens, the following numbers of samples were subjected to a complete IHC assessment of paired breast PTs and ALN metastatic tumors: 47 samples for ER, 44 samples for PR, 49 samples for HER2, 43 samples for Ki-67, 23 samples for E-cadherin, 44 samples for cyclin D1, 42 samples for Bcl-2, 50 samples for GCDFP-15, 21 samples for Snail+Slug, 22 samples for PD-L1, and 21 samples for PRL-3 [other reports on the heterogeneity of GCDFP-15 and PRL-3 expression in BC have been previously published elsewhere (35, 36)]. In total, 36 samples overlapped in ER, PR, and Ki-67 complete expression of PTs and ALN metastases, allowing both the primary BC surrogate intrinsic subtype and the shift in the matched metastasis to be determined. Among the PTs, 18 luminal-like subtypes were identified (luminal A-like: 12 cases, luminal B-like: six cases) and 18 other subtypes (in accordance with ESMO guidelines; explained in the *Materials and Methods* section). Among the matched ALN metastases, 17 luminal-like cases were identified (luminal A-like: 12 cases, luminal B-like: five cases) and 19 of another non-luminal subtype (HER2-positive, basal-like). Interestingly, the findings identified 11 cases with a switch in the surrogate intrinsic subtype between breast PT and its matched ALN metastasis; among these, five shifted from other to luminal-like subtype and six shifted from luminal-like to another subtype (**Table 2**).

**Table 2:** Distribution of the BC surrogate intrinsic subtypes, revealing a shift in subtype between PTs and synchronous ALN metastases.

	n = 36	
	Primary tumor	ALN metastases
Luminal	18	17
- A-like	12*	12*
- B-like	6	5
Other	18	19
Shift in	Primary tumor -> ALN metastasis	
Subtype		
- other -> luminal-like	5	
- luminal-like -> other	6	

*Nine cases showed consistent luminal A-like subtype in both paired PT and matched ALN metastasis.

ALN, axillary lymph node; PT, primary tumor.

The positive and negative expression rates between the PTs and their synchronous ALN metastases are shown in **Table 3**. Only the expression of E-cadherin differed significantly between PTs and matching ALN metastases (p = 0.0002). Moreover, significant differences were found in the ER index of positive cell nuclei (p = 0.05) and ER Allred score (p = 0.05) between PTs and matched ALN metastases (**Table 4**). Poor to substantial concordance was observed in expression between PTs and matching ALN lesions, as indicated by Cohen’s kappa coefficient (ER κ = 0.46*, PR κ = 0.54*, E-cadherin κ = 0, cyclin D1 κ = 0.28, Bcl-2 κ = 0.38*, GCDFP-15 κ = 0.28*, Snail+Slug κ = 0.64, PD-L1 κ = -0.12*, PRL-3 κ = -0.08; *p < 0.05; **Table 5**); however, HER2 demonstrated almost perfect concordance (κ = 1, p < 0.0001).

**Table 3:** The comparison of biomarker expression profiles in the breast PTs and ALN metastases presenting a total of positive and negative cases among PTs as well as positive and negative cases among ALN metastases.

Biomarker	Primary tumor	ALN metastases	p-value*
Ki-67			
Positive rate (%)	17/43 (39.5)	17/43 (39.5)	
Negative rate (%)	26/43 (60.5)	26/43 (60.5)	1
ER α			
Positive rate (%)	36/47 (76.6)	38/47 (80.8)	
Negative rate (%)	11/47 (23.4)	9/47 (19.1)	0.8
PR			
Positive rate (%)	18/44 (40.9)	22/44 (50)	
Negative rate (%)	25/44 (56.8)	22/44 (50)	0.58
HER2**			
Positive rate (%)	7/49 (14.3)	5/49 (10.2)	
Negative rate (%)	36/49 (73.5)	39/49 (79.6)	0.72
**E-cadherin**			
** Positive rate (%)**	**23/23 (100)**	**11/23 (47.8)**	
** Negative rate (%)**	**0/23 (0)**	**12/23 (52.2)**	**0.0002**
Cyclin D1			
Positive rate (%)	35/44 (79.5)	38/44 (86.4)	
Negative rate (%)	9/44 (20.5)	6/44 (13.6)	0.57
Bcl-2			
Positive rate (%)	28/42 (66.7)	31/42 (73.8)	
Negative rate (%)	14/42 (33.3)	11/42 (26.2)	0.63
GCDFP-15			
Positive rate (%)	31/50 (62)	31/50 (62)	
Negative rate (%)	19/50 (38)	19/50 (38)	1
PD-L1			
Positive rate (%)	3/22 (13.6)	2/22 (0.1)	
Negative rate (%)	19/22 (86.4)	20/22 (90.9)	1
Snail+Slug			
Positive rate (%)	19/21 (90.5)	20/21 (95.2)	
Negative rate (%)	2/21 (9.5)	1/21 (4.8)	1
PRL-3			
Positive rate (%)	16/21 (76.2)	14/21 (66.7)	
Negative rate (%)	5/21 (23.8)	7/21 (33.3)	0.73

*The analysis of the difference in biomarker expression status between breast PTs and ALN metastases was performed using χ^2^ test for Ki-67, ER α, PR, HER2, E-cadherin, cyclin D1, Bcl-2, GCDFP-15, and PRL-3. Due to low expected values (<5), the Fisher’s exact test was performed for PD-L1 and Snail+Slug analysis.

**The analysis was performed excluding cases of 2+ intensity score.

ALN, axillary lymph node; ER, estrogen receptor; PR, progesterone receptor; PT, primary tumor. Significant results are bolded.

**Table 4:** The numerical summary of numerical variables such as Ki-67 index of positive cell nuclei, ER, as well as PR index of positive cell nuclei and Allred score compared with paired Wilcoxon signed rank test.

Biomarker	Median (range)		Mean ± SD		p-value
	Primary tumor	ALN metastasis	Primary tumor	ALN metastasis	
Ki-67	11 (0–58)	10 (0–70)	16.1 ± 15.7	17.5 ± 17.8	0.47
**ER %**	**65 (0–100)**	**80 (0–100)**	**57 ± 36**	**66 ± 36**	**0.05**
**ER Allred**	**6 (0–8)**	**7 (0–8)**	**4.9 ± 2.99**	**5.7 ± 2.95**	**0.05**
PR %	10 (0–100)	25 (0–100)	29 ± 35	40 ± 42	0.07
PR Allred	3 (0–8)	3 (0–5)	3.05 ± 3.05	2.57 ± 2.3	0.17

ALN, axillary lymph node; ER, estrogen receptor; PR, progesterone receptor. Significant results are bolded.

**Table 5:** The Cohen’s kappa coefficient concordance analysis of expression status in breast PTs and synchronous ALN metastases.

Primary tumor	ALN metastases		
	0	1	Kappa
ER (n = 49)			0.46**
0	6	6	
1	3	34	
PR (n = 46)			0.54***
0	19	7	
1	3	15	
Ki-67 (n = 43)			0.51***
0	21	5	
1	5	12	
HER2 (n = 31^†^)			1*
0	27	0	
1	0	4	
E-cadherin (n = 23)			0
0	0	0	
1	12	11	
Cyclin D1 (n = 44)			0.28
0	3	6	
1	3	32	
Bcl-2 (n = 42)			0.38*
0	7	7	
1	4	25	
GCDFP-15 (n = 50)			0.28*
0	11	8	
1	9	22	
SNAIL+SLUG (n = 21)			0.64*
0	1	1	
1	0	19	
PD-L1 (n = 22)			-0.12*
0	17	2	
1	3	0	
PRL-3 (n = 21)			0.08
0	2	3	
1	5	11	

^†^The analysis was performed excluding cases of 2+ intensity score.

*p-value < 0.05.

**p-value < 0.001.

***p-value < 0.0001.

ALN, axillary lymph node; ER, estrogen receptor; PR, progesterone receptor; PT, primary tumor.

To support the above findings, the index of PT was found to moderately correlate with that in the matching ALN for Ki-67 (ρ = 0.55, p = 0.0004) and was strongly associated with PR expression (ρ = 0.79, p = 0.0004). The Ki-67 index negatively correlated also with the PR index among PTs (ρ = -0.76, p = 0.001), and the Ki-67 index in the ALN tumors negatively correlated with PR expression in PTs (ρ = -0.6, p = 0.02). In addition, a strong correlation in PR Allred scores was observed between PTs and the matching ALN lesions (ρ = 0.8, p = 0.0003). The analysis of intensity correlations among all biomarkers revealed a strong association of HER2 and Bcl-2 in primary and synchronous metastatic sites (ρ = 0.82, p = 0.0002; ρ = 0.75, p = 0.001, respectively), which is compliant with established concordance rates.

Among the PTs, positive correlations were observed between ER and Bcl-2 (ρ = 0.72, p = 0.002), ER and PRL-3 (ρ = 0.55, p = 0.03), Bcl-2 and PRL-3 (ρ = 0.57, p = 0.03), and Snail+Slug and PRL-3 (ρ = 0.61, p = 0.02). Among the ALN metastases, correlations were found between ER and PR (0.54, p = 0.04) and ER and cyclin D1 (ρ = 0.59, p = 0.02). Finally, correlations were also observed between PT Bcl-2 intensity and ALN metastatic HER2 (ρ = -0.53, p = 0.04), PT ER and ALN metastatic Bcl-2 (ρ = 0.78, p = 0.0006), PT PRL-3 and ALN metastatic Bcl-2 (ρ = 0.63, p = 0.01), and finally between PT cyclin D1 and ALN metastatic PRL-3 (ρ = -0.72, p = 0.002). Detailed results of Spearman’s correlation analysis are presented in **Supplementary Table S2**.

The following discordance rates in protein expression were observed between PTs and matched ALN metastases: Ki-67, 23.2%; ER, 17%; PR, 18.2%; HER2, none; E-cadherin, 52.2%; cyclin D1, 20.5%; Bcl-2, 26.2%; GCDFP-15, 30%; PD-L1, 22.7%; Snail+Slug, 4.8%, and PRL-3, 38.1% (**Table 6**). However, for the goal of the study, the most essential was complete loss or gain of immunoexpression in the metastatic foci. In total, in the ALN metastasis, Ki-67 expression demonstrated five losses and five gains, ER expression demonstrated three losses and five gains, while PR expression demonstrated two losses and six gains. Additionally, no changes in expression were observed for HER2; noteworthy, 18 samples scored 2+ were excluded from the study as equivocal, hence maintaining elusive relevance. E-cadherin expression was lost in 12 out of 23 ALN metastases with no gains. Finally, the ALN metastases demonstrated lower expression of cyclin D1 in three cases, Bcl-2 in four cases, GCDFP-15 in eight cases (35), PD-L1 in three cases, Snail+Slug in zero cases, and PRL-3 expression in five cases (36). On the other hand, the expression of the aforementioned biomarkers was gained in 6, 7, 7, 2, 1, and 3 cases, respectively.

**Table 6:** Discordance of the expression status of the biomarkers between breast PTs and synchronous ALN metastases.

Biomarker	Discordance rate (%)	ALN metastases	
		Loss of expression relative to the paired PT (+) -> (-)	Gain of expression relative to the paired PT (-) -> (+)
Ki-67	10/43 (23.2)	5	5
ER	8/47 (17)	3	5
PR	8/44 (18.2)	2	6
HER2	0/49	–	
E-cadherin	12/23 (52.2)	12	–
Cyclin D1	9/44 (20.5)	3	6
Bcl-2	11/42 (26.2)	4	7
GCDFP-15 (35)	15/50 (30)	8	7
PD-L1	5/22 (22.7)	3	2
Snail+Slug	1/21 (4.8)	–	1
PRL-3 (36)	8/21 (38.1)	5	3

It demonstrates how many paired samples showed the difference in the expression of the marker, i.e., how many ALN tumors gained or lost the expression of the marker relative to the paired PT sample.

ALN, axillary lymph node; ER, estrogen receptor; PR, progesterone receptor; PT, primary tumor.

**Figure 2:**
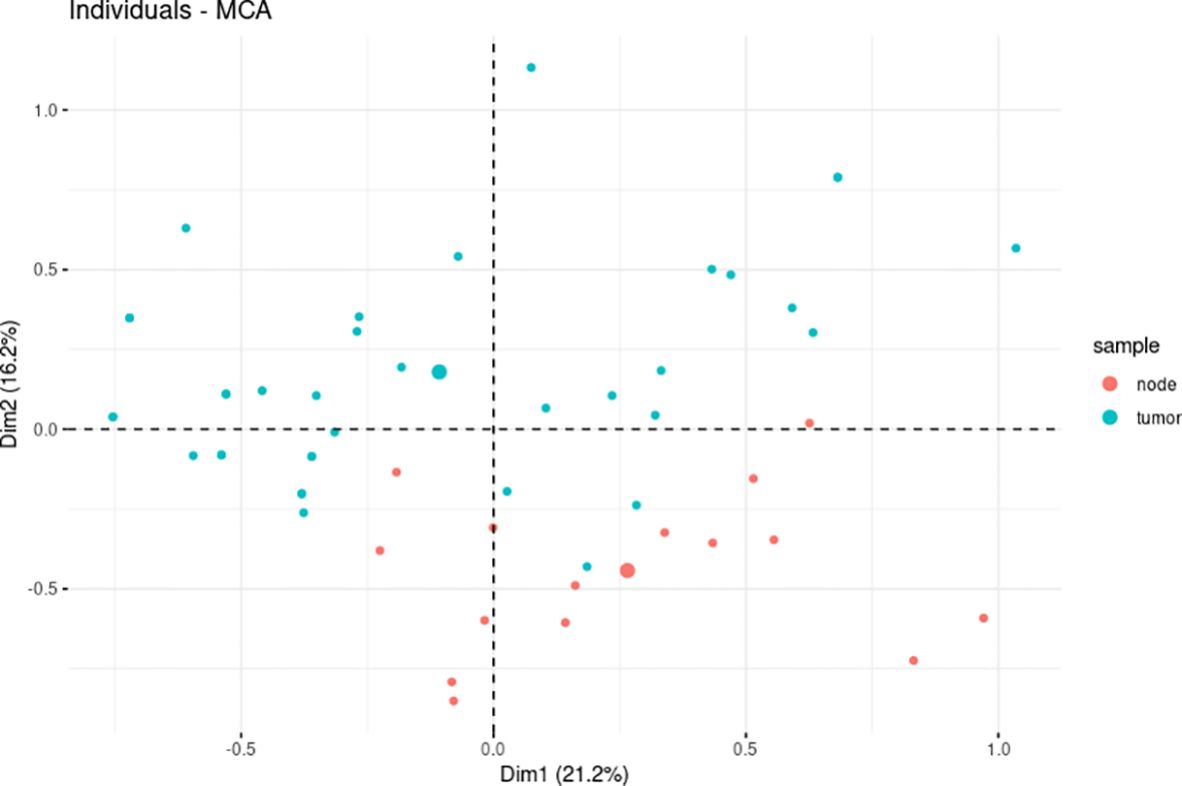
The multiple correspondence analysis (MCA) revealed divergent levels of estrogen receptor (ER), progesterone receptor (PR), HER2, Ki-67, Bcl-2, cyclin D1, E-cadherin, GCDFP-15, Snail+Slug, PD-L1, and PRL-3 expression between primary tumors (PTs) (n = 37) and synchronous axillary lymph node (ALN) metastases (n = 15).

The spatial partitioning of breast PTs and synchronous ALN metastases with regard to the resultant expression profile of all analyzed markers was determined using MCA. A total of 37 PT and 15 ALN metastatic samples, which demonstrated a complete set of markers, were tested. The PTs were found to have a distinct general expression profile to their matching ALN metastatic tumors. More specifically, total variance of 37.3% was found for the spatial differentiation of breast PTs from ALN metastases along with dimension 2. Interestingly, a few PT samples showed greater similarity to the ALN metastases, suggesting individual and disparate clinical characteristics (**Figure 2**).

## Discussion

One of the goals of modern oncology is the development of personalized medicine based on the molecular profile of BC. In BC, targeted therapies have been based on polyADP-ribose polymerase (PARP) inhibitors (BRCA1/2 receptor) (37) or anti-HER2-targeted drugs (38, 39). As increasing numbers of therapies restrict the subpopulations of BC clones, clonal homogeneity seems to be a key determinant of treatment efficacy. However, despite decades of study, the phenomenon of intertumoral heterogeneity in BC remains unclear, and such heterogeneity translates into therapeutic resistance and may cause improper disease management in some patients, as extensively described by Kalinowski et al. (40).

BC is frequently associated with metastases to the ALN, thus offering an opportunity to capture cellular subpopulations with more aggressive properties than those of the PTs. Synchronous assessment of the breast PTs and matched ALN metastases could therefore provide further insight into the required therapy type and modify clinical outcomes; however, this area is still poorly researched (41, 42). There is therefore a pressing need to reduce the likelihood of relapse in early BC due to residual survivor cells remaining after first-line treatment and to optimize subsequent therapies according to the cellular composition of metastatic tumors arising from the intertumoral heterogeneity.

To address this need, the present preliminary study compared the expression status of routinely used biomarkers (ER, PR, HER2, Ki-67) between PTs and matched ALN metastases. It also examined proteins known to be involved in the mechanisms of breast carcinogenesis and metastasis, as well as the related immunological response.

Previous studies on the differences in expression between breast PTs and synchronous metastases to ALNs have examined the degree of heterogeneity for well-known predictive biomarkers such as ER, PR, HER2, and Ki-67. Nedergaard et al. (43) report a 21% discrepancy in ER expression status between foci analyzed in 101 BC cases. Similarly, discordance values of 28.9%, 23.7%, and 8.9% for ER, PR, and HER2, respectively, were observed in a group of 190 BC cases (44), 8.8% and 11.3% discordance was observed for ER and HER2 expression in 80 cases of invasive ductal BC (45), and 28.8%, 31.7%, 13.5%, and 43.3% discordance in ER, PR, HER2, and Ki-67 expression was found between PTs and matching ALN metastases (46). In the previous studies, data about E-cadherin expression in both foci were divergent; it was identified in 95.45% of PTs and 72.73% of synchronous ALN metastases among 88 cases of BCs (47), concluding that part of the metastases lost the expression.

Moreover, in 49 non-lobular BCs, E-cadherin expression demonstrated poor consistency between the two foci (Cohen’s kappa κ = -0.040), but opposite to the previous study, ALN tumor displayed 18 cases of gain of expression and one case of loss of expression in comparison with the PT. Interesting results were obtained in 48 breast samples for master EMT regulators (TWIST1, SNAIL, SLUG) and classical BC receptors (ER, PR, HER2) at the mRNA and protein levels, as well as with disease-free survival (DFS) and overall survival (OS). Little agreement was observed between breast PT and ALN lesions for Snail and Slug (Snail: 76% positive cases in PTs and 45% in ALN tumors, κ = 0.06; Slug: 26% positive cases in PTs and 18% positive cases in ALN, κ = 0.18). In contrast, *SNAIL* expression demonstrated moderate agreement (55% and 48% positive cases in PTs and ALN tumors, accordingly, κ = 0.45). In addition, a negative-to-positive switch in Snail expression was correlated with worsened OS (HR = 4.6, p = 0.03) and DFS (HR = 3.8, p = 0.05) (48).

Many studies have demonstrated higher expression of PD-L1 in ALN lesions than in PT in cases of triple-negative BC correlating with high grade and decreased DFS (e.g., 49, 50); these observations played a role in the approval of a pembrolizumab-based anti-PD-L1 treatment for PD-L1-positive advanced BCs (21). However, due to biased PD-L1 assessment in early or low-rate TILs BC, there remains a need for routine PD-L1 status determination in both the PT and synchronous metastatic ALN.

Yuan et al. report significant differences in PD-L1 expression between PTs and ALN tumors, as well as associated PD-L1-positive metastatic lymph nodes with poor prognostic features, including higher Ki-67 index (p = 0.048), higher TNM stage (p = 0.012), and higher grade (p = 0.029). Nevertheless, they noted almost perfect concordance in PD-L1 expression status between the BC foci (85%) and did not find differences in the prevalence of PD-L1 between luminal A, luminal B, HER2-enriched, and triple-negative BC subtypes in either site (51).

Several reports indicate that various molecular biomarkers also demonstrate similar expression status in PT and metastatic lesions. For instance, the concordance rates of expression status in both foci have been found to reach 72.2% for ER, 88.9% for PR, and 90.7% for HER2 (52); rates of 77.6% for ER (κ = 0.534, p < 0.01), 82.2% for PR (κ = 0.640, p < 0.01), 84.1% for HER2 (κ = 0.647, p < 0.01) have also been reported in Chinese women (53), and rates of 96.7% for ER/PR (κ = 0.773, κ = 0.654, accordingly) and 90% for HER2 (κ = 0.785) have been recently reported (54).

A study of 10 biomarkers in the breast PTs and corresponding ALN metastases in 90 invasive ductal BCs found consistent expression of ER (r = 0.989, p < 0.000), PR (r = 0.989, p < 0.000), HER2 (r = 0.946, p < 0.000), Ki-67 (r = 0.918, p < 0.000), and Bcl-2 (r = 0.982, p < 0.000) in the PT and ALN foci accompanied by discordance rates of 3.4%, 3.9%, 4.4%, and 2.6% for ER/PR, HER2, Ki-67, and Bcl-2, respectively, as confirmed by the bivariate Pearson correlation (55). Markiewicz et al. (48) report substantial agreement for ER (57% and 70% positives in PTs and ALN tumors, κ = 0.63) and fair agreement for PR (63% and 78% positives in PTs and ALN tumors, κ = 0.31), as well as a very high agreement for HER2 (15% and 18% positives in PTs and ALN tumors, κ = 0.89) and E-cadherin expression (87% positives for both PTs and ALN tumors, κ = 1).

Our present findings are two-pronged. Moderate agreement between PTs and ALN metastases was observed for ER, PR, and Ki-67 expression (κ-values of 0.46, 0.54, and 0.51, respectively) with a switch in expression status, observed in approximately 20% of cases; however, HER2 demonstrated the same expression in both sites (**Table 5**). The BC surrogate intrinsic subtype was found to switch in 11 cases: six from luminal-like to another subtype and five from another type to a luminal-like BC surrogate intrinsic subtype among the ALN lesions (**Table 2**). The latter cases could benefit from the implementation of hormonal therapies, such as second-line treatment of BC.

In contrast, less agreement was found for E-cadherin, PD-L1, and PRL-3 between the foci, with κ-values of 0, -0.12, and 0.08, respectively. A loss of E-cadherin expression was observed in over 50% of ALN metastases with no gains. In addition, three losses and two gains (22.7%) were observed in the ALN tumor for PD-L1 and five losses and three gains (38.1%) for PRL-3 (**Tables 5** and **6**).

Substantial concordance between BC foci was observed for Snail and Slug, with only one gain of expression by the ALN tumor. This suggests the presence of ongoing EMT among the PTs and/or the maintenance of mesenchymal features to gain mobility; it also indicates the reverse process, i.e., mesenchymal-to-epithelial transition (MET), enabling the colonization of the new niche of the ALNs. These findings seem to indicate that clonal selection takes place within the PTs, enabling the acquisition of more aggressive and therapy-resistant features by the tumor cells.

Very little is known about the agreement between cyclin D1 and GCDFP-15 expression in the primary and synchronous ALN sites of BC, which emphasizes the need for further research beyond the standard area of well-known clinical biomarkers, as performed herein. The protein profiles characterizing the PTs and synchronous ALN metastases resulting from the MCA allowed the samples to be partitioned into two clusters with distinct features; this division justifies the expansion of standard diagnostic and prognostic procedures to include additional biomarkers, which may modify the decisions made upon treatment.

It may seem that the above-discussed results are evidence of intertumoral heterogeneity between PTs and matched ALN metastases; in fact, this is the affirmation of a much more disquieting problem of uncertain tumor assessment techniques used in clinical routine, on which we aimed to shed light. This preliminary study examines the clinical potential assessment of 11 IHC stains in treating BC. A substantial level of heterogeneity was observed, and this raises the question regarding the most accordant source of sampling tissue specimens for diagnostic use and prognosis as well as protocol for BC assessment and classification. The surrogate intrinsic subtypes being an adaptation of the primary intrinsic subtypes to clinical use are based on IHC staining (as recommended by ESMO), which would constitute an agreement between the low-cost technique and fairly high reliability of the assessment. Moreover, the presence of heterogeneity in ALN could influence clinical decisions. Elicitation of the expression of ER, PR, Ki-67, HER2, and PD-L1 would affect the possibility of using additional therapy, which may result in a more beneficial therapeutic outcome.

There are still many ambiguities that have not been adequately defined to this day such as area of the spot that should be examined, no defined cutoff values for Ki-67 in recommendations, the validity of the secondary assessment of the biopsy specimen collected after surgery, or simultaneous assessment of PT and ALN foci. It may be assumed that assessment of particular markers in the ALN metastases could offer greater therapeutic efficacy than approaches based solely on PT sampling from the preoperative biopsies. Additionally, there raises a question if preoperative biopsy, which has a diameter of a few millimeters, can be representative of the whole tumor for target therapy administration. Furthermore, reducing the rate of BC metastasis and relapse with proper personal therapy and better understanding of the relevance of the EMT-related proteins could increase the survival of BC patients.

Apart from the pilot character, this study highlights several novelties over currently available reports, which may be undoubtedly used as the seed for further research. It involves a wide spectrum of proteins, beyond the clinically, routinely used however reflects the standard assessment conditions of prognostic and predictive factors on preoperative biopsies. The majority of studies rather focus on heterogeneity in molecular markers, at the same time, omitting relevance of prognostic markers originally used in clinical examination. In turn, we combined the prognostic and molecular markers associated with EMT and its reversal, MET. On the other hand, like any other, our study has several limitations. Firstly, being only a pilot study, it is based on pathological examination alone, without follow-up data (further analyses are ongoing). In addition, it only uses one 3-mm core obtained from PT and the number of available samples was limited by technical issues during IHC staining. Furthermore, the HER2 status was not validated by FISH, and due to the short time from the beginning of the research on this patient group (2017), little follow-up data were collected. Lastly, the enrichment of the study with expression data would allow determining the actual molecular subtype of BC and hence casting a doubt on clinically used techniques of assessing the prognostic/predictive factors. Future studies should be performed on a larger cohort to confirm the trends as well as optimize the techniques employed by the clinicians to prognosticate and in the process of treatment decision-making. Nevertheless, the findings that we herein reported should be considered a prelude prompting to revise the current standards of BC assessment and classification. The assessment of the changes in molecular subtype in ALN and more precise evaluation of it may only imply the improvement of the patient therapeutic benefits; however, it requires further research.

## Data Availability Statement

The raw data supporting the conclusions of this article will be made available by the authors without undue reservation.

## Ethics Statement

The studies involving human participants were reviewed and approved by Research Ethics Committee of the Medical University of Lodz no. RNN/12/16/KE of January 19, 2016. The patients/participants provided their written informed consent to participate in this study.

## Author Contributions

Conceptualization, RK and KT. Data curation, WK and MO. Formal analysis, MO. Funding acquisition, RK and KT. Investigation, WK, MO, and ŁK. Methodology, WK, MO, and ŁK. Project administration, WK. Resources, WK, RK, and KT. Software, MO. Supervision, RK and KT. Validation, WK and ŁK. Visualization, MO. Writing—original draft, WK, MO, and ŁK. Writing—review and editing, RK and KT. All authors contributed to the article and approved the submitted version.

## Funding

The study was funded by the Medical University of Łódź grant no. 503/1-034-03/503-11-001.

## Conflict of Interest

The authors declare that the research was conducted in the absence of any commercial or financial relationships that could be construed as a potential conflict of interest.

## Publisher’s Note

All claims expressed in this article are solely those of the authors and do not necessarily represent those of their affiliated organizations, or those of the publisher, the editors and the reviewers. Any product that may be evaluated in this article, or claim that may be made by its manufacturer, is not guaranteed or endorsed by the publisher.
