# Supplementary material for: Intertumoral Heterogeneity of Primary Breast Tumors and Synchronous Axillary Lymph Node Metastases Reflected in IHC-Assessed Expression of Routine and Nonstandard Biomarkers
Source: Front Oncol. 2021 Nov 3;11:660318. doi: 10.3389/fonc.2021.660318 (PMC8595326; doi:10.3389/fonc.2021.660318)
Supplement: Supplementary file 1 [file DataSheet_1.docx]

Supplementary Material

# Supplementary Tables

Supplementary Table 1. The antibodies used in the study.

| **Antibody** | **Manufacturer** | **Code** | **Dilution** | **Clone** | **Incubation time** | **Antigen retrieval method** | **Control tissue** | **Criteria of expression assessment** |
| --- | --- | --- | --- | --- | --- | --- | --- | --- |
| **ER α** | Dako/Agilent | GA084 | Ready to use | EP1 | 30’ | PT link  pH 8.0  20’ | Endometrium | % index of positive cell nuclei,  Intensity assessment (0/1+/2+/3+) |
| **PR** |  | GA090 |  | PgR 1294 |  |  |  |  |
| **Ki-67** |  | GA626 |  | MIB-1 |  | PT link  pH 6.0  20’ | Lymph node | % index of positive cell nuclei |
| **HER2** |  | K5207 |  | - |  | Water bath  pH 6.0  40’ | Manufacturer’s control samples | Intensity assessment (0/1+/2+/3+) |
| **E-cadherin** |  | GA059 |  | NCH-38 |  | PT link  pH 8.0  20’ | Normal breast tissue | Presence/absence of expression (dummy var.) |
| **Bcl-2** |  | M0887 |  | 124 |  | Water bath  pH 9.0  20’ | Lymph node | Intensity assessment (0/1+/2+/3+) |
| **Cyclin D1** |  | GA083 |  | EP12 |  | PT link  pH 8.0  20’ | Tonsil |  |
| **GCDFP-15** |  | GA077 |  | 23A3 |  |  | Normal breast tissue |  |
| **Snail + Slug** | Abcam | ab180714 | 1:200 | polyclonal |  | PT link  pH 6.0  20’ | Normal renal tissue |  |
| **PD-L1** |  | ab213524 | 1:150 | EPR19759 | 60’ | Water bath  pH 9.0  30’ | Lymph node | Presence/absence of expression (dummy var.) |
| **PRL-3 (PTP4A3)** |  | ab50276 | 1:300 | polyclonal | 30’ | PT link  pH 8.0  20’ | Esophageal cancer | Intensity assessment (0/1+/2+/3+) |

Supplementary Table 2. Detailed statistics of Spearman's correlations of particular parameters.

|  |  | Spearman’s ρ | p-value |
| --- | --- | --- | --- |
| Index of postive cell nuclei | | | |
| **pt.Ki-67** | **aln.Ki-67** | **0.547896369967388** | **0.0344813755694657** |
| pt.Ki-67 | pt.ER% | -0.49459526837213 | 0.0608959854517477 |
| aln.Ki-67 | pt.ER% | -0.47057842126789 | 0.076674280187726 |
| pt.Ki-67 | aln.ER% | 0.107501294525007 | 0.702951906998448 |
| aln.Ki-67 | aln.ER% | -0.194051224731544 | 0.488316621800362 |
| pt.ER% | aln.ER% | 0.473015013379328 | 0.0749527191585306 |
| **pt.Ki-67** | **pt.PR%** | **-0.75690832271611** | **0.00108724069213739** |
| **aln.Ki-67** | **pt.PR%** | **-0.601590246641429** | **0.01766773929999** |
| pt.ER% | pt.PR% | 0.414835860051543 | 0.124165122910404 |
| aln.ER% | pt.PR% | 0.155091302738667 | 0.581007500399757 |
| pt.Ki-67 | aln.PR% | -0.489937640828755 | 0.0637528907798877 |
| aln.Ki-67 | aln.PR% | -0.372213266601417 | 0.171872815734922 |
| pt.ER% | aln.PR% | 0.405804753344968 | 0.133411290114237 |
| aln.ER% | aln.PR% | 0.222027215795812 | 0.426433395530511 |
| **pt.PR%** | **aln.PR%** | **0.789881658477174** | **0.000459513193330041** |
| Allred score | | | |
| pt.ER.Allred | aln.ER.Allred | 0.200561097695727 | 0.473549755471557 |
| pt.ER.Allred | pt.PR.Allred | 0.391318579291158 | 0.14920035925976 |
| aln.ER.Allred | pt.PR.Allred | 0.153435452343724 | 0.585100663535233 |
| pt.ER.Allred | aln.PR.Allred | 0.417737001652189 | 0.121290580755824 |
| aln.ER.Allred | aln.PR.Allred | 0.377443551120996 | 0.165453940608198 |
| **pt.PR.Allred** | **aln.PR.Allred** | **0.800333506340047** | **0.000338869153049703** |
| Intensity of expression | | | |
| pt.ER | aln.ER | 0.18475524766347 | 0.509774569106944 |
| pt.ER | pt.PR | 0.296967816947711 | 0.282438684281789 |
| aln.ER | pt.PR | 0.195544088800135 | 0.484911091686333 |
| pt.ER | aln.PR | 0.333767123896725 | 0.22406980908238 |
| **aln.ER** | **aln.PR** | **0.536600883487412** | **0.0391790634820297** |
| **pt.PR** | **aln.PR** | **0.512794342621932** | **0.0506206470270998** |
| pt.ER | pt.HER2 | -0.386020020236841 | 0.155275307199162 |
| aln.ER | pt.HER2 | 0.328777337432343 | 0.231499828345762 |
| pt.PR | pt.HER2 | 0.0103882998889829 | 0.970689268440498 |
| aln.PR | pt.HER2 | -0.00932955620356684 | 0.973675520954982 |
| pt.ER | aln.HER2 | -0.34794660573053 | 0.20378230632329 |
| aln.ER | aln.HER2 | 0.335642585286688 | 0.221316378674645 |
| pt.PR | aln.HER2 | -0.153895079321662 | 0.5839633126236 |
| aln.PR | aln.HER2 | -0.124482579148669 | 0.658475196567508 |
| **pt.HER2** | **aln.HER2** | **0.823014999600512** | **0.000164055814982689** |
| pt.ER | pt.cyclinD1 | 0.227964578880024 | 0.413842101660767 |
| aln.ER | pt.cyclinD1 | 0.016834984145632 | 0.952515137919428 |
| pt.PR | pt.cyclinD1 | 0.362551666125502 | 0.184155622791286 |
| aln.PR | pt.cyclinD1 | 0.0559773372214011 | 0.842931590691372 |
| pt.HER2 | pt.cyclinD1 | -0.0141414141414141 | 0.960106537722506 |
| aln.HER2 | pt.cyclinD1 | 0.0929576170347685 | 0.741774666978162 |
| pt.ER | aln.cyclinD1 | 0.0631949410915949 | 0.822958045094051 |
| **aln.ER** | **aln.cyclinD1** | **0.590224444378494** | **0.0205419152415027** |
| pt.PR | aln.cyclinD1 | 0.273579168576894 | 0.323819030056248 |
| aln.PR | aln.cyclinD1 | 0.450546796973303 | 0.0919135867117937 |
| pt.HER2 | aln.cyclinD1 | 0.0240012000900075 | 0.932338361431961 |
| aln.HER2 | aln.cyclinD1 | -0.106623183513437 | 0.705277748577992 |
| pt.cyclinD1 | aln.cyclinD1 | 0.0220011000825069 | 0.937966372951824 |
| **pt.ER** | **pt.Bcl-2** | **0.717631795207474** | **0.00259302919298698** |
| aln.ER | pt.Bcl-2 | -0.13065740797298 | 0.64255332815256 |
| pt.PR | pt.Bcl-2 | 0.176221622549168 | 0.529846250118892 |
| aln.PR | pt.Bcl-2 | 0.147052739755272 | 0.600987226030274 |
| pt.HER2 | pt.Bcl-2 | -0.353718779425362 | 0.195872249583596 |
| **aln.HER2** | **pt.Bcl-2** | **-0.534174738704773** | **0.0402473478995251** |
| pt.cyclinD1 | pt.Bcl-2 | 0.0110223982257195 | 0.968900925224659 |
| aln.cyclinD1 | pt.Bcl-2 | -0.0962303481482325 | 0.732984303582709 |
| **pt.ER** | **aln.Bcl-2** | **0.780487804878049** | **0.000595859733065307** |
| aln.ER | aln.Bcl-2 | 0.06158508255449 | 0.827404761551371 |
| pt.PR | aln.Bcl-2 | 0.280295939505032 | 0.311597481375771 |
| aln.PR | aln.Bcl-2 | 0.508448983132394 | 0.0529487776096498 |
| pt.HER2 | aln.Bcl-2 | -0.420468001045378 | 0.118626702629793 |
| aln.HER2 | aln.Bcl-2 | -0.469614209695127 | 0.0773632906466626 |
| pt.cyclinD1 | aln.Bcl-2 | 0.0719354893354743 | 0.798905392708646 |
| aln.cyclinD1 | aln.Bcl-2 | 0.242748821335968 | 0.383347190728983 |
| **pt.Bcl-2** | **aln.Bcl-2** | **0.75582508402804** | **0.00111593087972262** |
| pt.ER | pt.GCDFP-15 | 0.358664270771238 | 0.189254555351954 |
| aln.ER | pt.GCDFP-15 | -0.11586430264935 | 0.680925817959503 |
| pt.PR | pt.GCDFP-15 | 0.225426107590928 | 0.419201549444795 |
| aln.PR | pt.GCDFP-15 | 0.0176224950511818 | 0.950296351825628 |
| pt.HER2 | pt.GCDFP-15 | 0.0282828282828283 | 0.9203007458646 |
| aln.HER2 | pt.GCDFP-15 | 0.0419442906132492 | 0.882011076848704 |
| pt.cyclinD1 | pt.GCDFP-15 | -0.213131313131313 | 0.445660139988083 |
| aln.cyclinD1 | pt.GCDFP-15 | 0.123006150461288 | 0.662302748794826 |
| pt.Bcl-2 | pt.GCDFP-15 | 0.293596607285074 | 0.288198936616445 |
| aln.Bcl02 | pt.GCDFP-15 | 0.377914612987774 | 0.1648837243143 |
| pt.ER | aln.GCDFP-15 | 0.0829945913727977 | 0.76871366835061 |
| aln.ER | aln.GCDFP-15 | 0.139206177827848 | 0.620747629585281 |
| pt.PR | aln.GCDFP-15 | -0.00420227421382749 | 0.988141263263962 |
| aln.PR | aln.GCDFP-15 | -0.0199182874460782 | 0.943830020677608 |
| pt.HER2 | aln.GCDFP-15 | 0.285002669132189 | 0.303195082402575 |
| aln.HER2 | aln.GCDFP-15 | 0.147890663925877 | 0.598891897939033 |
| pt.cyclinD1 | aln.GCDFP-15 | -0.0480112023269278 | 0.865079730217618 |
| aln.cyclinD1 | aln.GCDFP-15 | 0.392403940641168 | 0.147975926271784 |
| pt.Bcl-2 | aln.GCDFP-15 | 0.172271030798888 | 0.539256072730202 |
| aln.Bcl02 | aln.GCDFP-15 | 0.125004199351621 | 0.65712480062232 |
| **pt.GCDFP-15** | **aln.GCDFP-15** | **0.52199413593745** | **0.0459411521482402** |
| pt.ER | pt.SNAIL+SLUG | 0.407496014552502 | 0.131645203850468 |
| aln.ER | pt.SNAIL+SLUG | 0.136111216953756 | 0.62860945039542 |
| pt.PR | pt.SNAIL+SLUG | -0.125227165925326 | 0.656547873699905 |
| aln.PR | pt.SNAIL+SLUG | 0.484659654712066 | 0.0671063575260786 |
| pt.HER2 | pt.SNAIL+SLUG | -0.202560660126346 | 0.469057811946096 |
| aln.HER2 | pt.SNAIL+SLUG | -0.189017990184259 | 0.499881374475736 |
| pt.cyclinD1 | pt.SNAIL+SLUG | -0.410811226436016 | 0.128229638693521 |
| aln.cyclinD1 | pt.SNAIL+SLUG | -0.203925054305611 | 0.4660046935997 |
| pt.Bcl-2 | pt.SNAIL+SLUG | 0.283352748600882 | 0.306125286716259 |
| aln.Bcl02 | pt.SNAIL+SLUG | 0.43945648628211 | 0.101212438747645 |
| pt.GCDFP-15 | pt.SNAIL+SLUG | 0.0147937560766432 | 0.958267700458793 |
| aln.GCDFP-15 | pt.SNAIL+SLUG | -0.103575617088102 | 0.713368491119411 |
| pt.ER | aln.SNAIL+SLUG | 0.349215147884789 | 0.202026691067353 |
| aln.ER | aln.SNAIL+SLUG | 0.255996055794858 | 0.357085743424667 |
| pt.PR | aln.SNAIL+SLUG | 0.21483446221183 | 0.441945937865119 |
| aln.PR | aln.SNAIL+SLUG | 0.214376880303149 | 0.44294229212297 |
| pt.HER2 | aln.SNAIL+SLUG | 0.0522232967867094 | 0.853356275620586 |
| aln.HER2 | aln.SNAIL+SLUG | 0.136756316403799 | 0.626967675912551 |
| pt.cyclinD1 | aln.SNAIL+SLUG | -0.226300952742407 | 0.417350460466147 |
| aln.cyclinD1 | aln.SNAIL+SLUG | -0.120641847820499 | 0.668448214382019 |
| pt.Bcl-2 | aln.SNAIL+SLUG | 0.310838025875246 | 0.259467656290645 |
| aln.Bcl02 | aln.SNAIL+SLUG | 0.33175439049055 | 0.227048601499813 |
| pt.GCDFP-15 | aln.SNAIL+SLUG | 0.295932015124686 | 0.284201163224303 |
| aln.GCDFP-15 | aln.SNAIL+SLUG | -0.369693864865253 | 0.17502234856655 |
| pt.SNAIL+SLUG | aln.SNAIL+SLUG | 0.156892908110547 | 0.576567481403576 |
| pt.ER | pt.PRL-3 | 0.547114989312058 | 0.0347920323678435 |
| aln.ER | pt.PRL-3 | 0.089126386653346 | 0.752102821167582 |
| pt.PR | pt.PRL-3 | 0.280484097002537 | 0.311259021922424 |
| aln.PR | pt.PRL-3 | 0.393914595261711 | 0.146282957933811 |
| pt.HER2 | pt.PRL-3 | -0.444444444444444 | 0.0969522857439344 |
| aln.HER2 | pt.PRL-3 | -0.374097727091142 | 0.169541543510866 |
| pt.cyclinD1 | pt.PRL-3 | -0.297979797979798 | 0.28072304253654 |
| aln.cyclinD1 | pt.PRL-3 | -0.0700035002625219 | 0.804208262535938 |
| **pt.Bcl-2** | **pt.PRL-3** | **0.571160635332737** | **0.0261492710513289** |
| **aln.Bcl-2** | **pt.PRL-3** | **0.628169061802733** | **0.0121512261859777** |
| pt.GCDFP-15 | pt.PRL-3 | 0.232323232323232 | 0.404723493593873 |
| aln.GCDFP-15 | pt.PRL-3 | 0.00510757471563062 | 0.985586745425413 |
| **pt.SNAIL+SLUG** | **pt.PRL-3** | **0.608819961615703** | **0.0160073130644403** |
| aln.SNAIL+SLUG | pt.PRL-3 | 0.435194139889245 | 0.104955184898725 |
| pt.ER | aln.PRL-3 | -0.296318878994877 | 0.283542127544978 |
| aln.ER | aln.PRL-3 | -0.148835827541189 | 0.596531907786683 |
| pt.PR | aln.PRL-3 | -0.204657747098158 | 0.464369154413935 |
| aln.PR | aln.PRL-3 | -0.162114244805305 | 0.563779872897109 |
| pt.HER2 | aln.PRL-3 | 0.311831777147643 | 0.257866937269518 |
| aln.HER2 | aln.PRL-3 | 0.0943989063492377 | 0.737899729006259 |
| **pt.cyclinD1** | **aln.PRL-3** | **-0.718033697379441** | **0.00257190061795498** |
| aln.cyclinD1 | aln.PRL-3 | 0.0751511128924708 | 0.790097396071672 |
| pt.Bcl-2 | aln.PRL-3 | -0.0203514514765765 | 0.942610323904475 |
| aln.Bcl-2 | aln.PRL-3 | -0.141986129518379 | 0.613718288114271 |
| pt.GCDFP-15 | aln.PRL-3 | 0.324140926245576 | 0.238539759982449 |
| aln.GCDFP-15 | aln.PRL-3 | 0.172200652548973 | 0.539424369367299 |
| pt.SNAIL+SLUG | aln.PRL-3 | 0.00462250163521024 | 0.986955478275691 |
| aln.SNAIL+SLUG | aln.PRL-3 | 0.106066017177982 | 0.706754766230341 |
| pt.PRL-3 | aln.PRL-3 | -0.0923186182344996 | 0.743494478667221 |

# Supplementary Figures


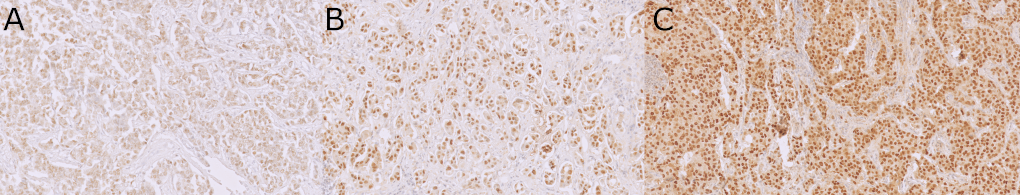


**Supplementary Figure 1. Immunohistochemistry staining for ER of A) 1+, B) 2+, and C) 3+ score.**


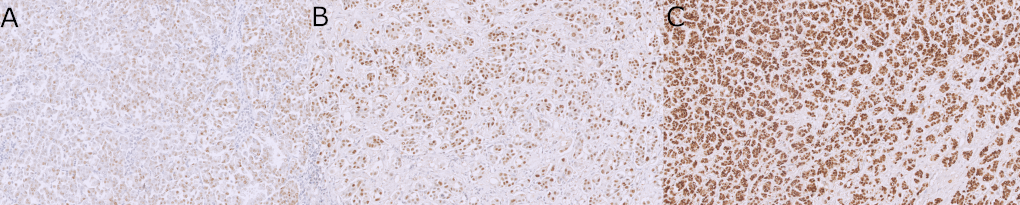


**Supplementary Figure 2. Immunohistochemistry staining for PR of A) 1+, B) 2+, and C) 3+ score.**


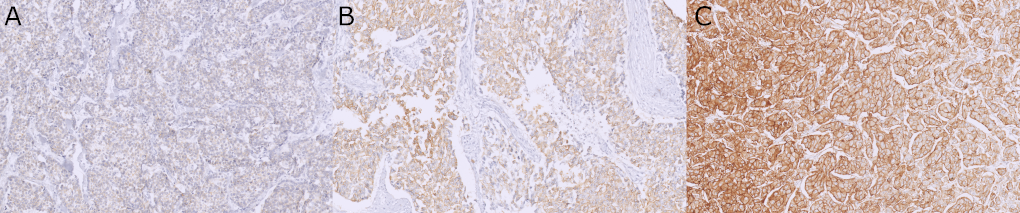


**Supplementary Figure 3. Immunohistochemistry staining for HER2 of A) 1+, B) 2+, and C) 3+ score.**


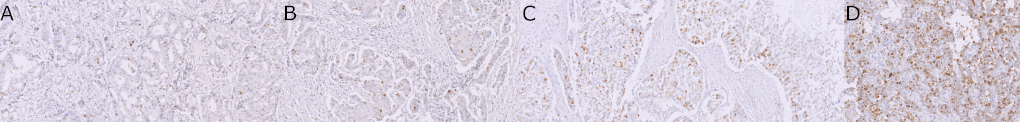


**Supplementary Figure 4. Ki-67 immunohistochemistry staining: A) 5% positive tumor cells, B) 10% positive tumor cells, C) 20% positive tumor cells, and D) 40% positive tumor cells.**


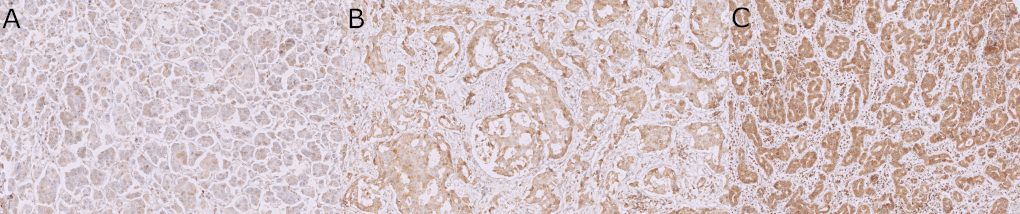


**Supplementary Figure 5. Immunohistochemistry staining for Bcl-2 of A) 1+, B) 2+, and C) 3+ score.**


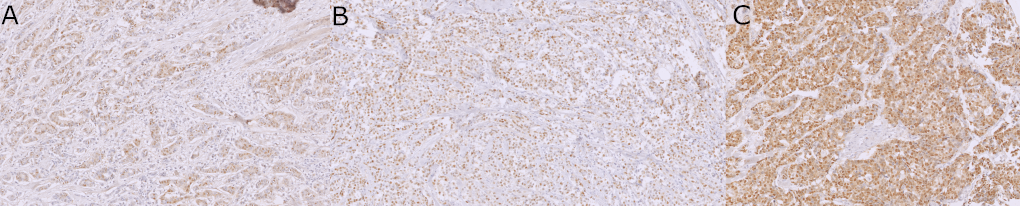


**Supplementary Figure 6. Immunohistochemistry staining for cyclin D1 of A) 1+, B) 2+, and C) 3+ score.**


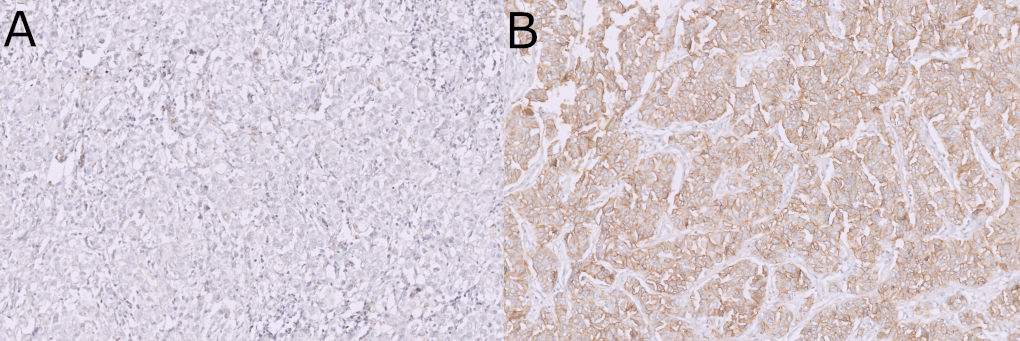


**Supplementary Figure 7. Immunohistochemistry staining for E-cadherin: A) negative, B) positive.**


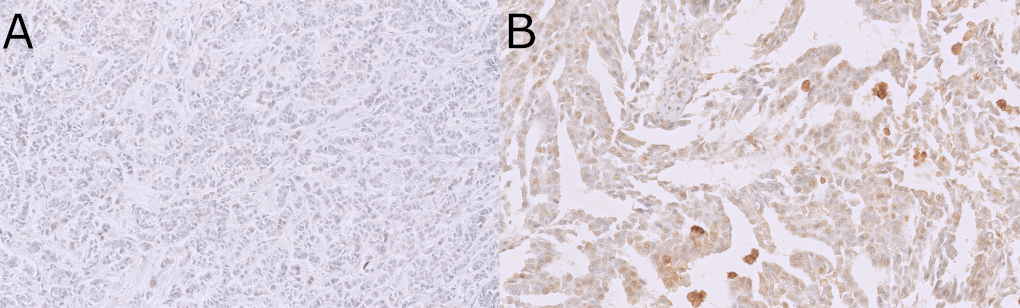


**Supplementary Figure 8. Immunohistochemistry staining for PD-L1: A) negative, B) positive.**


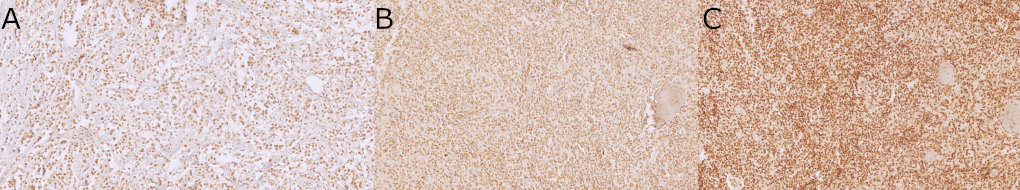


**Supplementary Figure 9. Immunohistochemistry staining for Snail+Slug (mix) of A) 1+, B) 2+, and C) 3+ score.**

Immunohistochemistry staining scans for GCDFP-15 and PRL-3 may be found in the previous articles:

Kuncman W, Orzechowska M, Taran K, Kordek R. Ekspresja genu kodującego gross cystic disease fluid protein 15 (GCDFP-15) w pierwotnych i przerzutowych ogniskach raka piersi. *Państwo i Społeczeństwo* (2019) **4**: 27–39. doi:10.34697/2451-0858-PIS-2019-4-002

Kuncman W, Orzechowska M, Taran K, Kordek R. Ewaluacja ekspresji fosfatazy tyrozynowej 4A3 (protein tyrosine phosphatase 4A3, PRL-3) – nieznane aspekty heterogenności pierwotnych ognisk raka piersi. *Państwo i Społeczeństwo* (2019) **4**: 41–55. doi:10.34697/2451-0858-PIS-2019-4-003
